# Evaluation of the Recovery Is Possible for Everyone transdiagnostic treatment and support program for adults with eating disorders

**DOI:** 10.1186/2050-2974-1-S1-O2

**Published:** 2013-11-14

**Authors:** Sarah Harry, Fiona Sutherland, Bree McCarthy, Karen Richards, Leah Brennan

**Affiliations:** 1 Recovery is Possible for Everyone, Australia; 2 Monash University School of Psychology and Psychiatry, Australia; 3 Australian Catholic University School of Psychology, Australia

## 

This research program examined the effectiveness of the Recovery is Possible for Everyone(RIPE) a community based transdiagnostic group treatment program for adults with eating disorders.

Study 1 used retrospectively collected clinical data to evaluate the effectiveness of two versions of the RIPE program, the original CBT-based program and a more recent ACT-based program. Pre and post EAT26 scores improved for both groups (n=97, *p*<.05); there were no difference between the CBT and ACT versions of the program (*p*>.05). High levels of client satisfaction and clinically significant reductions in binging and purging were reported (n=100).

Study 2 used prospectively evaluated the effectiveness of the RIPE program (ACT-based). Participants (n=15; 100% female; M=25.7y; BMI=17.1- 28.7kg/m2) reported elevated levels of eating disorder and general psychopathology (*p*< .05). Weight preoccupation and drive for thinness, dietary restraint, internally responsive eating, body image satisfaction, self-esteem and quality of life improved significantly (*p* < .05). Interpretations are limited by the small sample size.

This is one of the first studies evaluating ACT based eating disorder treatment and one of the few studies evaluating the 'real world' effectiveness of eating disorder intervention. Results provide preliminary evidence for the effectiveness of ACT in the treatment for eating disorders. This study highlights the viability of proving evidence based intervention and conducting practice based research in real world settings.

This abstract was presented in the **Adult Treatment and Services** stream of the 2013 ANZAED Conference.

